# Factors associated with positive cancer screening for the uterine cervix and breast in Jakarta Province, Indonesia: a cross-sectional study

**DOI:** 10.1186/s12885-022-10381-1

**Published:** 2022-12-14

**Authors:** Lady Margaretha Febriany Sirait, Nobuyuki Hamajima, Yunosuke Suzuki, Endang Sri Wahyuningsih, Dwi Oktavia, Widyastuti, Souphalak Inthaphatha, Kimihiro Nishino, Eiko Yamamoto

**Affiliations:** 1 Department of Healthcare Administration, Nagoya University Graduate School of Medicine, 65 Tsurumai-cho, Showa-ku, 466-8550 Nagoya, Japan; 2 Jakarta Department of Health, DKI Jakarta Provincial Government, Jalan Kesehatan Raya No. 10, Kecamatan Gambir Kota Jakarta Pusat, DKI Jakarta, Indonesia

**Keywords:** Breast cancer, Cancer screening, Cervical cancer, Indonesia, Population-based, Risk factors

## Abstract

**Background:**

In many middle-income countries, cancer incidence and mortality are rapidly increasing, but data for developing a strategy of cancer control are rarely collected or analyzed. This study aimed to identify factors associated with positive cancer screening for the uterine cervix and breast in Jakarta Province, Indonesia.

**Methods:**

The data of 79,660 women who had visual inspection with acetic acid (VIA) and 83,043 women who had clinical breast examination (CBE) in the Jakarta Women Cancer Screening program in 2019 were included in this study. Socio-demographic factors, reproductive factors, lifestyle factors, family history, and the results of VIA and CBE were used for analyses. Binary and multivariate logistic regression analyses were performed to identify factors associated with VIA positive or CBE positive.

**Results:**

The positive rate was 0.9% for both VIA and CBE among the screening participants. Factors associated with VIA positive were age < 30 years old, age at menarche ≤ 11 years old, remarriage, lower educational level, having an occupation, partner’s occupation other than being an employee, alcohol consumption, smoker, inadequate physical activity, cancer family history, and no Pap smear history. Factors associated with CBE positive were age at menarche ≤ 11 years old, widowed, high education, having an occupation, no breastfeeding history, birth control history, alcohol consumption, smoker, inadequate physical activity, cancer family history, and breast tumor history.

**Conclusion:**

Factors associated with VIA positive and CBE positive among Indonesian women were revealed. To promote female cancer prevention in Indonesia, the prevalence of screenings should be increased and education about the risk factors should be provided to medical professionals.

**Supplementary Information:**

The online version contains supplementary material available at 10.1186/s12885-022-10381-1.

## Introduction

Cancer was the second leading cause of death in the world in 2018, but most cancer deaths occur in low- and middle-income countries [1]. Cancer incidence and cancer mortality are rising rapidly in middle-income countries, and the proportion of advanced cancers and the case fatality rate are higher compared to high-income countries [1–4]. Prevention and early detection are the major strategies for cancer control, but the data for these strategies, such as the incidence, risk factors, outcomes, and stages at diagnosis, are not revealed in most middle-income countries.

In low- and middle-income countries, cervical cancer is the leading cause of female cancer death and the incidence of cervical cancer was the highest besides breast cancer in several countries [1, 5]. Papanicolaou (Pap) smear is the most common screening method for cervical cancer worldwide and human papillomavirus (HPV) vaccination is the most effective preventive method [6, 7]. However, visual inspection using acetic acid (VIA) is used in places with limited resources [8], where a Pap smear or an HPV test are not available. A meta-analysis including 29 studies reported that the sensitivity and specificity of VIA for cervical intraepithelial neoplasia (CIN) grade 2 or worse were 73.2% and 86.7%, respectively [9]. For screening breast cancer, clinical breast examination (CBE) is used instead of mammography in developing countries [10]. CBE contributes to down-staging of 17–47% of breast cancers from the advanced stage to the early stage [11]. Previous studies reported that the sensitivity and specificity of CBE is 50–54% and 94–98%, respectively [12–14]. VIA and CBE are non-invasive and cheap methods and the results of VIA and CBE can be available immediately after examinations [15, 16]. Therefore, VIA and CBE are alternative screening methods in low- and middle-income countries. In Asia, VIA is used in the cervical cancer screening program in Bangladesh [17], Thailand [18], India [19], and China [20] and CBE is the first choice for breast cancer screening in low- and middle-income countries [10, 21].

In Indonesia, a middle-income country in Southeast Asia, the most common cancer in women is breast cancer followed by cervical cancer [1, 22]. A screening program using VIA and CBE was introduced in 2007 and developed into a national program in 2015 to provide cancer screening services widely and increase cancer awareness [15, 23]. There are some studies on VIA or CBE that included women at hospitals or civil servants in Indonesia [23–26]. The studies reported that the VIA positive rate was 1.4–4.7% and that the risk factors for positive VIA were the number of marriages, parity, smoking, and less use of hormonal contraception [23, 24]. Wahidin et al. reported the coverage, the positive rate, and the suspected cancer prevalence in cervical and breast cancer screening program in Indonesia [27]. However, to date there has been no population-based study on risk factors of female cancers in Indonesia. This study aimed to identify the factors associated with positive cancer screening for the uterine cervix and breast using the data of the national screening program in Jakarta Province in 2019.

## Methods

### Study design and participants

**Fig. 1:**
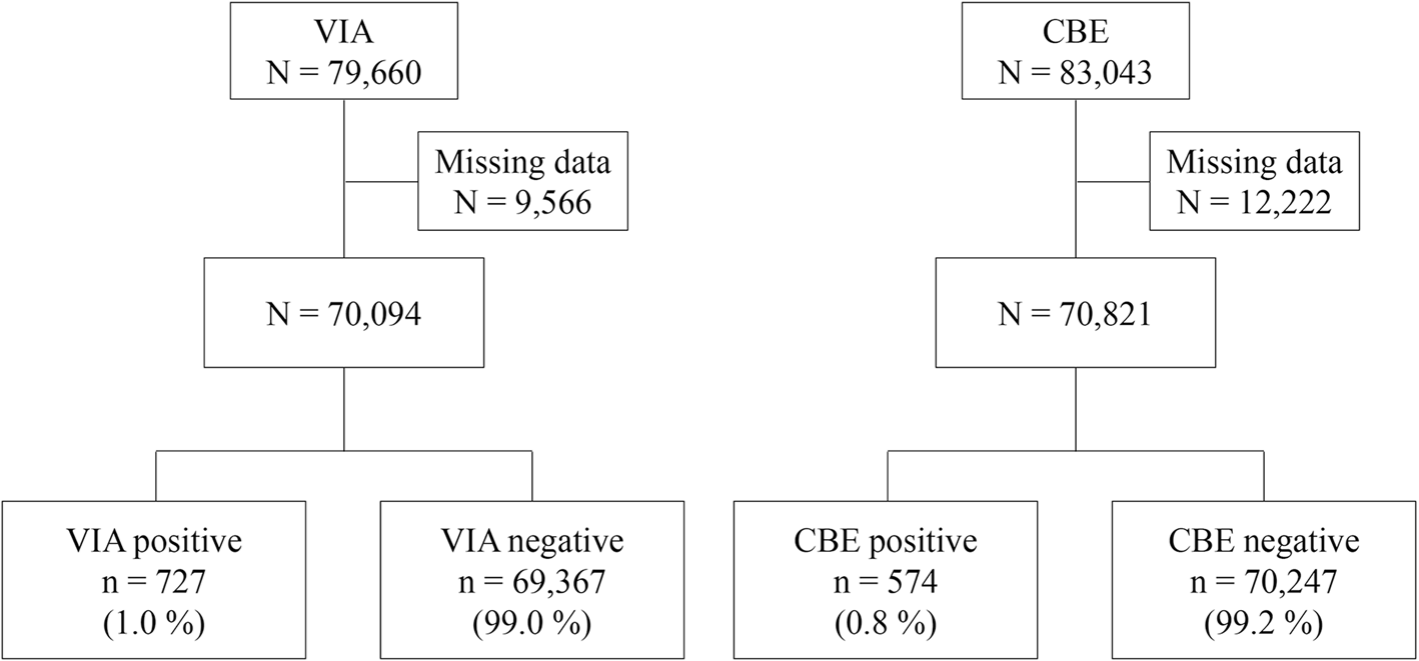
Flow diagram of participants of this study. This study included 70,094 of 79,660 women who had VIA and 70,821 of 83,043 women who had CBE. VIA, visual inspection with acetic acid; CBE, clinical breast examination

This study is a cross-sectional study and used the secondary data of all female citizens of Jakarta who had VIA and/or CBE in 2019, which were taken from the Jakarta Women Cancer Screening Database in Jakarta Department of Health. Women who reached menarche, had sexual intercourse, were not pregnant, had an intact uterus, and had no history of CIN or cervical cancer were eligible for VIA. Women who had menarche, were not pregnant, and were not receiving treatment for breast cancer could have CBE. Women had VIA and CBE when they had no menstrual bleeding. Written informed consent for VIA, CBE, and cryotherapy was obtained after a complete explanation in conjunction with asking several questions related to their conditions by a doctor using “the women cancer early detection procedure form” (Additional file 1). Women had VIA and/or CBE at 334 public health centers or in specific places, such as village schools or community centers, by mobile screening teams on mutually suitable dates. Three clinics that were supported by the University of Indonesia and the Indonesian Cancer Foundation also provided screenings to women. In 2019, the total female population was 5,272,489 in the province and the number of target women (30–50 years old) was estimated as 1,665,148. In fact, 79,660 women had VIA, 83,043 women had CBE, and 78,934 women underwent both VIA and CBE. The coverage of VIA and CBE was estimated to be 5.0% and 4.8%, respectively. The medical records of 9,566 women who had VIA and 12,222 women who had CBE had missing data regarding the 15 variables that were used in the statistical analyses and the women were excluded in this study. Finally, we included 70,094 women who had VIA and 70,821 women who had CBE in the study (Fig. 1).

### VIA

A doctor with two midwives or nurses formed a team and conducted VIA. After applying 3–5% acetic acid to the cervix for a minute, the cervix was observed. The result was positive when an acetowhite area was found in the transformation zone, negative when no change was observed, and suspicion of invasive cancer when a growth or ulcerative lesion was found. When a woman had a positive result, doctors provided cryotherapy after obtaining written informed consent from her husband. When the result was suspicion of invasive cancer, the woman was referred to a hospital. The maximum number of VIA tests per day by a team was 35 to ensure the quality of tests.

### CBE

CBE was performed by female doctors or nurses who underwent CBE training to recognize different types of abnormalities and warning signs in the breasts [28]. The training was provided by the Ministry of Health or the Jakarta Department of Health. When an abnormal breast mass was detected or palpable, the woman was referred to a hospital for mammography or ultrasonography.

### Data collection

The data of all women who underwent VIA and/or CBE were sent from public health centers and the three clinics to the Jakarta Department of Health and saved in the Jakarta Women Cancer Screening Database. For this study, the results of VIA and CBE and the following 15 variables were derived from the database: age, age at menarche, marital status, partner’s marital status, educational level, occupation, partner’s occupation, alcohol consumption, smoking habit, daily physical activity, history of breastfeeding, birth control history, Pap smear history, breast tumor history, and the family history of cancer. Age was divided into four groups with 10 years intervals. Based on a previous study that showed that most Indonesian girls reached menarche at 12–14 years old [29], the age of menarche was divided into three groups: 9–11 years, 12–14 years, and 15–17 years. Marital status was categorized into three groups: single/first marriage, remarriage, and widowed. The educational level was divided into three groups: low (none and elementary school), middle (junior high school and high school), and high (bachelor and master degrees). Occupation was categorized into two groups (housewife and other), because most respondents were housewives. Partner’s occupation was also divided into two groups (employee and other) and others included traders, army, manual labor, and others. Alcohol consumption was defined as having one drink or more per day. Regarding smoking, smokers and passive smokers were classified into “yes.” The daily physical activity was set at 30 min per day based on the World Health Organization recommendation that adults should perform at least 150–300 min of moderate-intensity aerobic physical activity throughout a week for substantial health benefits [30]. Birth control history was defined as having an experience of using contraception methods, such as an intrauterine device, condom, pill, shot, and implant.

### Statistical analysis

Data were analyzed using Statistical Package for Social Sciences, version 27 (IBM SPSS Inc, Armonk, NY, USA). Logistic regression analyses were performed to obtain odds ratio (OR) and 95% confidence interval (CI). In multivariable analyses on VIA positive and CBE positive, the forced-entry method was used including all the 15 variables. Statistical significance was set at P < 0.05. The Hosmer-Lemeshow test was performed to assess the fit of the logistic regression model and the results indicated a good fit of the model for the data to identify factors associated with VIA positive (*P* = 0.776) and CBE positive (*P* = 0.354).

### Ethical considerations

This study was approved by the Ethics Committee of Nagoya University Graduate School of Medicine (approval number 2021 − 0130). Written informed consent was taken from each woman who had VIA and/or CBE in the program. All methods were performed in accordance with the relevant guidelines and regulations.

## Results

### Number of tests and results in the women cancer screening program in Jakarta, 2019

In 2019, 79,660 and 83,043 women underwent VIA tests and CBEs, respectively (Table 1). The number of target women of the program in Jakarta Province was 1,665,148. Of 79,660 women, 756 women (0.9%) were positive for VIA, including 74 women who were strongly suspected of having invasive cervical cancer. On the other hand, 753 women (0.9%) of 83,043 women showed positive CBE findings. Women who underwent cryotherapy accounted for only 24.9% (*n* = 188) of women who were positive for VIA. Most women (99.3%) underwent tests at public health centers or in their communities. East Jakarta had the highest number of screening tests. Women who underwent screenings at the clinics had the highest positive rate of both VIA (8.1%) and CBE (5.2%).

**Table 1:** Number of tests and results in the women cancer screening program

	Cervical cancer screening			Breast cancer screening	
	VIA	Positive (%)	Cryotherapy	CBE	Positive (%)
Public health centers					
Central Jakarta	10,220	168 (1.6%)	92	10,478	140 (1.3%)
North Jakarta	10,114	128 (1.3%)	26	10,156	54 (0.5%)
West Jakarta	14,843	52 (0.4%)	4	15,094	80 (0.5%)
South Jakarta	10,293	61 (0.6%)	10	10,302	102 (1.0%)
East Jakarta	32,352	276 (0.9%)	45	32,490	208 (0.6%)
Thousand islands	1,331	30 (2.2%)	0	1,331	4 (0.3%)
Clinics^a^	507	41 (8.1%)	11	3,192	165 (5.2%)
Total	79,660	756 (0.9%)	188	83,043	753 (0.9%)

*VIA *visual inspection with acetic acid, *CBE *clinical breast examination

^a^Three clinics supported by University of Indonesia and Indonesian Cancer Foundation

### Characteristics of women who had VIA and CBE

The data of 70,094 VIA and 70,821 CBEs were analyzed in this study. Most women were aged 40–49 years (VIA, 35.3%; CBE, 35.2%), reached menarche at 12–14 years old (VIA, 83.6%; CBE 83.6%), and were single or married for the first time (VIA, 93.3%; CBE, 93.3%) (Table 2). The main educational level was the middle level (VIA, 65.4%; CBE, 65.2%), followed by the high level (VIA, 24.6%; CBE, 24.8%). Most women were housewives (VIA, 76.0%; CBE, 75.9%). Regarding lifestyle factors, most women did not consume more than one alcoholic drink per day (VIA and CBE, 99,1%) and were not smokers (VIA, 90.5%; CBE, 90.4%). Only 25.3% (both VIA and CBE) had daily physical activity totaling more than 30 min. Most respondents had a history of breastfeeding (VIA, 67.7%; CBE, 67.6%) and had used birth control methods in their lives (VIA, 62.0%; CBE 61.7%). Only 11.2% of women had a Pap smear in both of the groups of VIA and CBE. Women with a history of breast tumors and cancer in family members were 1.1% and 1.2%, respectively.

**Table 2:** Characteristics of women who took VIA and CBE in the screening program

Characteristics	VIA (*N*=70,094)		CBE (*N*=70,821)	
	N	%	N	%
Age (years old)				
<30	10,434	14.9	10,656	15.1
30-39	21,759	31.1	21,918	30.9
40-49	24,755	35.3	24,931	35.2
≥50	13,146	18.7	13,316	18.8
Age at menarche (years old)				
9-11	3,654	5.2	3,691	5.2
12-14	58,589	83.6	59,217	83.6
15-17	7,851	11.2	7,913	11.2
Marital status				
Single	420	0.6	695	1.0
First marriage	64,989	92.7	65,358	92.3
Remarriage	2,864	4.1	2,909	4.1
Widowed	1,821	2.6	1,859	2.6
Partner’s marital status^a^				
First marriage	67,121	95.8	67,529	95.3
Remarriage	2,553	3.6	2,597	3.7
NA	420	0.6	695	1.0
Educational level				
High	17,287	24.6	17,592	24.8
Middle	45,820	65.4	46,137	65.2
Low	6,987	10.0	7,092	10.0
Occupation				
Housewife	53,311	76.0	53,729	75.9
Other	16,783	24.0	17,092	24.1
Partner’s occupation				
Employee	25,826	36.8	26,047	36.8
Other	44,268	63.2	44,774	63.2
Alcohol consumption				
No	69,452	99.1	70,169	99.1
Yes	642	0.9	652	0.9
Smoking^b^				
No	63,404	90.5	64,015	90.4
Yes	6,690	9.5	6,806	9.6
Daily physical activity				
No	52,387	74.7	52,914	74.7
Yes	17,707	25.3	17,907	25.3
Breastfeeding history				
No	22,608	32.3	22,957	32.4
Yes	47,486	67.7	47,864	67.6
Birth control history				
No	26,642	38.0	27,118	38.3
Yes	43,452	62.0	43,703	61.7
Pap smear history				
No	62,228	88.8	62,893	88.8
Yes	7,866	11.2	7,928	11.2
Breast tumor history				
No	69,278	98.9	69,988	98.8
Yes	816	1.1	833	1.2
Cancer family history				
No	69,288	98.8	69,983	98.8
Yes	806	1.2	838	1.2

*VIA* visual inspection with acetic acid, *CBE* clinical breast examination, *NA* not applicable; Pap, Papanicolaou

^a^Answers of single women were not applicable

^b^Smoking includes passive smokers

### Factors associated with VIA positive

Of the 70,094 women, 727 (1.0%) were positive for VIA. Binary and multivariate logistic regression analyses were performed on positive VIA (Table 3). In binary logistic regression analysis, women who were 30–39 years old and ≥50 years old (than < 30 years old), and had a birth control history had significantly less VIA positive (Table 3). Reaching menarche at 9–11 years old (rather than 15–17 years), remarriage (rather than single/first marriage), low and middle educational level (rather than high educational level), alcohol consumption, smoker, inadequate daily physical activity, no history of breastfeeding, and cancer family history were associated with positive VIA. Multivariate analyses adjusted by all variables in Table 3 showed that factors associated with positive VIA were menarche at 9–11 years old (compared to 15–17 years), remarriage (compared to single/first marriage), a low educational level (compared to a high level), having a job, husband’s job other than employee, alcohol consumption, smoker, inadequate physical activity, no Pap smear history, and a cancer family history. Women in the age groups of 30–39 years and ≥ 50 years were significantly less likely to be VIA positive than those in the age group < 30 years.

**Table 3:** Odds ratio and 95% confidence interval of VIA positive among 70,094 women

Variables	Positive	OR (95% CI)	*P*	AOR (95% CI)	*P*
	N (%)				
Age (years old)					
<30	137 (1.3%)	1 (Reference)		1 (Reference)	
30-39	215 (1.0%)	0.75 (0.60-0.93)	0.009	0.76 (0.61-0.95)	0.015
40-49	268 (1.1%)	0.82 (0.67-1.01)	0.065	0.83 (0.67-1.03)	0.085
≥50	107 (0.8%)	0.61 (0.48-0.79)	<0.001	0.60 (0.46-0.79)	<0.001
Age at menarche (years old)					
9-11	85 (2.3%)	2.37 (1.74-3.24)	<0.001	2.39 (1.75-3.26)	<0.001
12-14	564 (0.9%)	0.97 (0.76-1.23)	0.793	0.95 (0.75-1.22)	0.709
15-17	78 (1.0%)	1 (Reference)		1 (Reference)	
Marital status					
Single/first marriage	658 (1.0%)	1 (Reference)		1 (Reference)	
Remarriage	44 (1.5%)	1.53 (1.13-2.09)	0.006	1.69 (1.15-2.40)	0.006
Widowed	25 (1.4%)	1.37 (0.92-2.05)	0.125	1.20 (0.79-1.81)	0.380
Partner’s marital status^a^					
First marriage	698 (1.0%)	1 (Reference)		1 (Reference)	
Remarriage	29 (1.1%)	1.10 (0.76-1.60)	0.616	0.72 (0.46-1.12)	0.147
Educational level					
High	151 (0.9%)	1 (Reference)		1 (Reference)	
Middle	487 (1.1%)	1.22 (1.01-1.47)	0.034	1.22 (0.99-1.49)	0.051
Low	89 (1.3%)	1.47 (1.12-1.91)	0.005	1.45 (1.14-2.02)	0.009
Occupation					
Housewife	532 (1.0%)	1 (Reference)		1 (Reference)	
Other	195 (1.2%)	1.16 (0.99-1.37)	0.068	1.31 (1.09-1.56)	0.003
Partner’s occupation					
Employee	253 (1.0%)	1 (Reference)		1 (Reference)	
Other	474 (1.1%)	1.09 (0.94-1.27)	0.251	1.20 (1.01-1.41)	0.029
Alcohol consumption					
No	704 (1.0%)	1 (Reference)		1 (Reference)	
Yes	23 (3.6%)	3.63 (2.37-5.54)	<0.001	3.46 (2.17-5.49)	<0.001
Smoking^b^					
No	640 (1.0%)	1 (Reference)		1 (Reference)	
Yes	87 (1.3%)	1.29 (1.03-1.62)	0.026	1.29 (1.01-1.65)	0.037
Daily physical activity					
No	653 (1.2%)	3.01 (2.36-3.83)	<0.001	3.69 (2.85-4.79)	<0.001
Yes	74 (0.4%)	1 (Reference)		1 (Reference)	
Breastfeeding history					
No	284 (1.3%)	1.35 (1.16-1.57)	<0.001	1.11 (0.93-1.32)	0.260
Yes	443 (0.9%)	1 (Reference)		1 (Reference)	
Birth control history					
No	315 (1.2%)	1 (Reference)		1 (Reference)	
Yes	412 (0.9%)	0.80 (0.69-0.93)	0.003	0.89 (0.77-1.05)	0.178
Pap smear history					
No	656 (1.0%)	1.17 (0.91-1.50)	0.212	1.33 (1.03-1.71)	0.028
Yes	71 (0.9%)	1 (Reference)		1 (Reference)	
Breast tumor history					
No	716 (1.0%)	1 (Reference)		1 (Reference)	
Yes	11 (1.3%)	1.30 (0.71-2.39)	0.379	1.05 (0.57-1.93)	0.874
Cancer family history					
No	691 (1.0%)	1 (Reference)		1 (Reference)	
Yes	36 (4.5%)	4.64 (3.29-6.53)	<0.001	6.34 (4.42-9.08)	<0.001

*VIA* visual inspection with acetic acid, *OR* odds ratio, *CI* confidence interval, *AOR* adjusted odds ratio, *Pap* Papanicolaou

^a^Partner’s marital status of single women are included in “first marriage.”

^b^Smoking includes passive smokers

### Factors associated with CBE positive

Of the 70,821 women, 574 women (0.8%) were positive for CBE. Binary logistic regression analysis showed that menarche at 9–11 years old (rather than 15–17 years old), widowed (rather than single/first marriage), occupation other than a housewife, alcohol consumption, smoker, inadequate daily physical activity, birth control history, breast tumor history, and cancer family history were associated with positive CBE (Table 4). Women who had a low or middle educational level (rather than the high level) were significantly less likely to have CBE positive. In multivariate analysis, reaching menarche at 9–11 years old (rather than 15–17 years old), widowed (rather than single/first marriage), occupation other than housewife, alcohol consumption, smoker, inadequate daily physical activity, no history of breastfeeding, birth control history, history of breast tumor, and family history of cancer were associated with CBE positive (Table 4). The low or middle educational level (rather than high level) was associated with less CBE positive.

**Table 4:** Odds ratio and 95% confidence interval of CBE positive among 70,821 women

Variables	Positive	OR (95% CI)	*P*	AOR (95% CI)	*P*
	N (%)				
Age (years old)					
<30	91 (0.8%)	1 (Reference)		1 (Reference)	
30-39	192 (0.9%)	1.03 (0.79-1.32)	0.841	1.08 (0.84-1.40)	0.534
40-49	194 (0.8%)	0.91 (0.71-1.17)	0.462	0.97 (0.75-1.25)	0.815
≥50	97 (0.7%)	0.85 (0.64-1.13)	0.274	0.94 (0.69-1.26)	0.672
Age at menarche (years old)					
9-11	56 (1.5%)	1.70 (1.20-2.42)	0.003	1.63 (1.14-2.32)	0.008
12-14	447 (0.8%)	0.84 (0.65-1.08)	0.174	0.81 (0.63-1.05)	0.109
15-17	71 (0.9%)	1 (Reference)		1 (Reference)	
Marital status					
Single/first marriage	521 (0.8%)	1 (Reference)		1 (Reference)	
Remarriage	27 (0.9%)	1.18 (0.79-1.74)	0.408	1.08 (0.69-1.71)	0.719
Widowed	26 (1.4%)	1.78 (1.20-2.65)	0.004	1.51 (1.01-2.29)	0.049
Partner’s marital status^a^					
First marriage	553 (0.8%)	1 (Reference)		1 (Reference)	
Remarriage	21 (0.8%)	1.00 (0.64-1.54)	0.991	0.79 (0.47-1.32)	0.374
Educational level					
High	240 (1.4%)	1 (Reference)		1 (Reference)	
Middle	282 (0.6%)	0.44 (0.37-0.53)	<0.001	0.44 (0.36-0.54)	<0.001
Low	52 (0.7%)	0.53 (0.39-0.72)	<0.001	0.54 (0.39-0.74)	<0.001
Occupation					
Housewife	388 (0.7%)	1 (Reference)		1 (Reference)	
Other	186 (1.0%)	1.51 (1.27-1.80)	<0.001	1.25 (1.04-1.51)	0.018
Partner’s occupation					
Employee	215 (0.8%)	1 (Reference)		1 (Reference)	
Other	359 (1.1%)	0.97 (0.82-1.15)	0.735	1.16 (0.97-1.39)	0.106
Alcohol consumption					
No	559 (0.8%)	1 (Reference)		1 (Reference)	
Yes	15 (2.3%)	2.93 (1.74-4.92)	<0.001	1.78 (1.02-3.11)	0.042
Smoking^b^					
No	457 (0.7%)	1 (Reference)		1 (Reference)	
Yes	117 (1.7%)	2.43 (1.98-2.98)	<0.001	2.39 (1.92-2.98)	<0.001
Daily physical activity					
No	451 (0.8%)	1.24 (1.02-1.52)	0.033	1.63 (1.31-2.03)	<0.001
Yes	123 (0.7%)	1 (Reference)		1 (Reference)	
Breastfeeding history					
No	184 (0.8%)	0.98 (0.82-1.17)	0.853	1.26 (1.02-1.56)	0.033
Yes	390 (0.8%)	1 (Reference)		1 (Reference)	
Birth control history					
No	186 (0.7%)	1 (Reference)		1 (Reference)	
Yes	388 (0.9%)	1.29 (1.09-1.55)	0.004	1.22 (1.02-1.47)	0.031
Pap smear history					
No	495 (0.8%)	0.79 (0.62-1.00)	0.051	0.96 (0.75-1.23)	0.736
Yes	79 (1.0%)	1 (Reference)		1 (Reference)	
Breast tumor history					
No	521 (0.7%)	1 (Reference)		1 (Reference)	
Yes	53 (6.4%)	9.06 (6.77-12.12)	<0.001	7.98 (5.84-10.91)	<0.001
Cancer family history					
No	530 (0.8%)	1 (Reference)		1 (Reference)	
Yes	44 (5.2%)	7.26 (5.29-9.95)	<0.001	4.29 (3.05-6.03)	<0.001

*CBE* clinical breast examination, *OR* odds ratio, *CI* confidence interval, *AOR* adjusted odds ratio, *Pap* Papanicolaou

^a^Partner’s marital status of single women are included in “first marriage.”

^b^Smoking includes passive smokers

## Discussion

This study showed that both the VIA positive rate and the CBE positive rate were 0.9% in the Jakarta Women Cancer Screening Program in 2019. The positive rate in this study was lower than the average rate of VIA positive (3.4%) and CBE positive (5.4%) in the whole country from 2007 to 2018 but the positive rate varied among provinces (VIA, 0.6–8.9%; CBE, 0–17.2%) [27]. The positive rate can be different depending on the characteristics of women who had screening. There have been two previous studies on the VIA positive rate in Jakarta. One study included patients of the university hospital in Jakarta from 2007 to 2011 and the positive rate was 4.7% [23]. Another study used the data of cancer screening for female civil servants and wives of civil servants in Jakarta, October 2017 and the positive rate was 1.4% [26]. This study showed that the positive rate in the population-based screening was lower compared to that in the hospital-based or working place-based screening. Compared to a population-based screening, more detail information of clinical data and outcome can be collected but a positive rate can be higher in a hospital-based screening because a hospital has better medical resources and patients have some symptoms. In order to make a policy for the general population in a region or a country, results of population-based screening are more important and needed.

The CBE positive rate in Jakarta women was lower in this study than that in 2007–2008, which was 14.2% among 1,179 women [25]. The reason for the lower CBE positive rate in this study may be because this study included more women < 40 years old (46.0%) than the previous study (18.4%). In the previous study, 14 women (1.2%) of the 1,179 women were finally diagnosed as having breast cancer by ultrasound and tissue sampling [25]. Of the 14 women diagnosed as having breast cancer, all were mammography positive and 13 were CBE positive (CBE was performed by trained nurses and midwives). Of the women who were CBE positive, 41.3% were mammography positive and 7.8% were diagnosed as having breast cancer [25]. These results suggest that CBE by trained nurses and midwives is useful for the screening for breast cancer. However, a further study on the final diagnosis of breast cancer among women with CBE positive in the population-based screening program is needed to evaluate the effectiveness of CBE in the program.

This study showed socio-demographic and reproductive risk factors of cervical cancer and breast cancer in Indonesia, other than lifestyle risk factors and family cancer history. Alcohol consumption, smoker, and inadequate physical activity have already been reported as risk factors of many cancers and healthy lifestyle is recommended in cancer prevention strategies [7, 31]. Women who had menarche at a young age (9–11 years) and birth control history were associated with positive CBE. The hormonal risk factors for breast cancer are caused by the biological mechanisms and have a synergistic effect with higher education, less exercise, higher body mass index (BMI), and alcohol consumption [32, 33]. However, the socio-demographic characteristics of a population are different according to the economic status of countries. To prevent breast cancer, therefore, it is important to identify the risk factors in each country, especially low- and middle-income countries [34]. Furthermore, in middle-income countries, patient navigation services in cancer care are not well established at most health facilities, which are needed to reduce advanced cancers, poor access to affordable and high-quality treatment, and preventable cancer deaths [35, 36]. The data related to risk factors of cancers are needed for education to healthcare providers in communities in order to reduce early delays in diagnosis as well as to support patients through their treatment [37].

Factors associated with cervical cancer screening positive are different among Asian countries; older age, lower educational level, lower socioeconomic status, higher parity in Bangladesh [17]; coitarche, years of sexual activity, low BMI, multiple partner in Thailand [38]; older age (> 40 years old), post-menopause, and smoking in rural China [39]; and younger age (< 30 years old), early marriage (18–<21 years old), and early birth age in India [40]. A systematic study including 90 papers reported that factors associated with breast cancers in Asia are age, early menarche, late menopause, nulliparity, positive family history, excessive fat consumption, alcohol, and smoking [41]. These results in previous studies are almost consistent with the results in our study except for age. It is reported that the peak of annual incidence of CIN I is 20–24 years old and CIN II/III is 25–29 years old [42] and that CIN of younger patients is more regressed compared to that of older patients [43]. Therefore, younger age may be associated with cervical cancer screening positive when the screening method is VIA.

In this study, 11.2% of women had had a Pap smear and they had a lower VIA positive rate than the others. This result may suggest that women with a Pap smear history have already had treatment for CIN or cervicitis. In the female cancer screening program in Indonesia, cryotherapy is provided to women when they are found to be positive for VIA, although their husband’s consent is needed. One of benefits of VIA is providing the result and cryotherapy at the same time and place as the VIA is performed. When more women have VIA in the screening program, the VIA positive rate may decrease by providing more treatment for VIA positive women. On the other hand, there were 1.2% of women who had breast tumor history, which was associated with CBE positive. A previous study conducted in 2016 showed that the prevalence of breast cancer screening was 18.7% in Indonesia and having a screening was associated with the level of knowledge of breast cancer’s risk factors, signs, and symptoms [44]. In this study, the estimated prevalence of VIA and CBE was 4.8% and 5.0%, respectively. To prevent female cancers more efficiently, the female cancer screening program is effective, but the screening prevalence should be increased through education and public awareness [37, 45].

There are some limitations to this study. First, this is an observational study and it might have selection bias, such as non-response bias, and information bias, such as reporting bias and recall bias. This study could not include some variables that have been reported as risk factors for cervical cancer or breast cancer, such as parity, gravidity, and BMI [7, 46, 47], because there were many missing data on these variables. Data were manually entered into the forms for the women cancer program database. Therefore, error reporting could easily occur. The results of this study may change when these variables are included in the analyses or the data are collected or entered correctly. Second, the results of further examinations and the final diagnoses in women who were positive for VIA and CBE were not available in this study. To evaluate the effectiveness of VIA and CBE in the screening program, this information is needed. A system for following positive cases in the screening program needs to be established. Third, the findings may not be representative of Indonesia’s entire population, because this study was conducted only in the Jakarta Province.

## Conclusion

The positive rate was 0.9% for both VIA and CBE among the screening participants in Jakarta Province. Other than lifestyle factors and cancer family history, socio-demographic and reproductive factors that were associated with VIA positive and CBE positive were identified. To promote cancer prevention and the early detection of female cancer in Indonesia, the prevalence of screenings should be increased and education concerning risk factors should be provided to medical professionals.

## Electronic supplementary material

Additional file 1 Women cancer early detection procedure form.

## Data Availability

The data that support the findings of this study are available from the Jakarta Department of Health but restrictions apply to the availability of these data, which were used under license for the current study, and therefore are not publicly available. Data are however available from the authors upon reasonable request and with permission of the Jakarta Department of Health.

## Abbreviations

BMI: Body mass index
CBE: Clinical breast examination
CI: Confidence interval
CIN: Cervical intraepithelial neoplasia
HPV: Human papillomavirus
OR: Odds ratio
Pap: Papanicolaou
VIA: Visual inspection with acetic acid

## References

[1] Bray F, Ferlay J, Soerjomataram I, Siegel RL, Torre LA, Jemal A (2018). Global cancer statistics 2018: GLOBOCAN estimates of incidence and mortality worldwide for 36 cancers in 185 countries. CA Cancer J Clin.

[2] Ngwa W, Olver I, Schmeler KM (2020). The use of health-related technology to reduce the gap between developed and undeveloped regions around the Globe. Am Soc Clin Oncol Educ Book.

[3] Ferlay J, Colombet M, Soerjomataram I, Mathers C, Parkin DM, Pineros M (2019). Estimating the global cancer incidence and mortality in 2018: GLOBOCAN sources and methods. Int J Cancer.

[4] Allemani C, Matsuda T, Di Carlo V, Harewood R, Matz M, Niksic M (2018). Global surveillance of trends in cancer survival 2000-14 (CONCORD-3): analysis of individual records for 37 513 025 patients diagnosed with one of 18 cancers from 322 population-based registries in 71 countries. Lancet.

[5] Torre LA, Islami F, Siegel RL, Ward EM, Jemal A (2017). Global Cancer in women: burden and trends. Cancer Epidemiol Biomarkers Prev.

[6] Rerucha CM, Caro RJ, Wheeler VL (2018). Cervical Cancer screening. Am Fam Physician.

[7] Tergas AI, Wright JD (2019). Cancer prevention strategies for women. Obstet Gynecol.

[8] Gupta R, Gupta S, Mehrotra R, Sodhani P (2017). Cervical Cancer screening in resource-constrained countries: current status and future directions. Asian Pac J Cancer Prev.

[9] Qiao L, Li B, Long M, Wang X, Wang A, Zhang G (2015). Accuracy of visual inspection with acetic acid and with Lugol’s iodine for cervical cancer screening: Meta-analysis. J Obstet Gynaecol Res.

[10] da Costa Vieira RA, Biller G, Uemura G, Ruiz CA, Curado MP (2017). Breast cancer screening in developing countries. Clin (Sao Paulo).

[11] Ngan TT, Nguyen NTQ, Van Minh H, Donnelly M, O’Neill C (2020). Effectiveness of clinical breast examination as a ‘stand-alone’ screening modality: an overview of systematic reviews. BMC Cancer.

[12] Albert US, Schulz KD (2003). Clinical breast examination: what can be recommended for its use to detect breast cancer in countries with limited resources?. Breast J.

[13] Eddy DM (1989). Screening for breast cancer. Ann Intern Med.

[14] Barton MB, Harris R, Fletcher SW (1999). The rational clinical examination. Does this patient have breast cancer? The screening clinical breast examination: should it be done?. How? JAMA.

[15] University of Indonesia Strategic Studies Grant Recipient Team (2018). Five Pillar Model in the implementation of Cervical Cancer Prevention Programs. Tim Penerima Hibah Kajian Strategis Universitas Indonesia.

[16] Denny L, Quinn M, Sankaranarayanan R (2006). Chapter 8: screening for cervical cancer in developing countries. Vaccine.

[17] Nessa A, Ara R, Fatema P, Nasrin B, Chowdhury A, Khan KH (2020). Influence of demographic and Reproductive factors on cervical pre-cancer and Cancer in Bangladesh. Asian Pac J Cancer Prev.

[18] Ploysawang P, Rojanamatin J, Prapakorn S, Jamsri P, Pangmuang P, Seeda K (2021). National cervical Cancer screening in Thailand. Asian Pac J Cancer Prev.

[19] Adsul P, Manjunath N, Srinivas V, Arun A, Madhivanan P (2017). Implementing community-based cervical cancer screening programs using visual inspection with acetic acid in India: a systematic review. Cancer Epidemiol.

[20] Hu SY, Zhao XL, Zhao FH, Wei LH, Zhou Q, Niyazi M, et al. Implementation of visual inspection with acetic acid and Lugol’s iodine for cervical cancer screening in rural China. Int J Gynaecol Obstet. 2022;1–8. 10.1002/ijgo.14368. 10.1002/ijgo.14368 35871356

[21] Newman LA (2022). Breast cancer screening in low and middle-income countries. Best Pract Res Clin Obstet Gynaecol.

[22] WHO. Indonesia (Source: Globocan 2020). 2021. Available from: https://gco.iarc.fr/today/data/factsheets/populations/360-indonesia-fact-sheets.pdf. Cited 2021 June 21.

[23] Nuranna L, Donny NB, Purwoto G, Winarto H, Utami TW, Anggraeni TD (2017). Prevalence, age distribution, and risk factors of Visual Inspection with acetic acid-positive from 2007 to 2011 in Jakarta. J Cancer Prev.

[24] Nuranna L, Aziz MF, Cornain S, Purwoto G, Purbadi S, Budiningsih S (2012). Cervical cancer prevention program in Jakarta, Indonesia: see and treat model in developing country. J Gynecol Oncol.

[25] Kardinah D, Anderson BO, Duggan C, Ali IA, Thomas DB (2014). Short report: limited effectiveness of screening mammography in addition to clinical breast examination by trained nurse midwives in rural Jakarta, Indonesia. Int J Cancer.

[26] Sirait LM, Anggreni LA, Pradibta CA, Widyastuti W, Prasetyo WE, Puspitasari HD (2019). Prevalence and factors associated with visual inspection of the cervix after acetic acid application positive result among female civil servants and wives of civil servants in Jakarta. Future Healthc J.

[27] Wahidin M, Febrianti R, Susanty F, Hasanah SR (2022). Twelve years implementation of cervical and breast Cancer screening program in Indonesia. Asian Pac J Cancer Prev.

[28] Veitch D, Goossens R, Owen H, Veitch J, Molenbroek J, Bochner M (2019). Evaluation of conventional training in clinical breast examination (CBE). Work.

[29] Batubara JR, Soesanti F, van de Waal HD (2010). Age at menarche in indonesian girls: a national survey. Acta Med Indones.

[30] WHO (2020). Guidelines on physical activity and sedentary Behaviour.

[31] Bray F, Soerjomataram I. The Changing Global Burden of Cancer: Transitions in Human Development and Implications for Cancer Prevention and Control. In: Gelband H, Jha P, Sankaranarayanan R, Horton S, editors. Cancer: Disease Control Priorities, Third Edition (Volume 3). Washington (DC): World Bank; 2015. 26913347

[32] Coughlin SS (2019). Epidemiology of breast Cancer in women. Adv Exp Med Biol.

[33] Tamimi RM, Spiegelman D, Smith-Warner SA, Wang M, Pazaris M, Willett WC (2016). Population attributable risk of modifiable and nonmodifiable breast Cancer risk factors in postmenopausal breast Cancer. Am J Epidemiol.

[34] McCormack VA, Boffetta P (2011). Today’s lifestyles, tomorrow’s cancers: trends in lifestyle risk factors for cancer in low- and middle-income countries. Ann Oncol.

[35] Dalton M, Holzman E, Erwin E, Michelen S, Rositch AF, Kumar S (2019). Patient navigation services for cancer care in low-and middle-income countries: a scoping review. PLoS ONE.

[36] Freeman HP, Rodriguez RL (2011). History and principles of patient navigation. Cancer.

[37] Dare AJ, Knapp GC, Romanoff A, Olasehinde O, Famurewa OC, Komolafe AO (2021). High-burden cancers in Middle-income countries: a review of Prevention and early detection strategies targeting At-risk populations. Cancer Prev Res (Phila).

[38] Lertcharernrit J, Sananpanichkul P, Suknikhom W, Bhamarapravatana K, Suwannarurk K, Leaungsomnapa Y (2016). Prevalence and Risk Assessment of Cervical Cancer Screening by Papanicolaou Smear and Visual Inspection with Acetic acid for pregnant women at a thai Provincial Hospital. Asian Pac J Cancer Prev.

[39] Zhang Q, Xie W, Wang F, Li RH, Cui L, Wang H (2016). Epidemiological investigation and risk factors for cervical lesions: cervical Cancer screening among women in rural Areas of Henan Province China. Med Sci Monit.

[40] Vidhubala E, Shewade HD, Niraimathi AK, Ramkumar S, Ramaswamy G, Nagalekshmi G (2019). Call for systematic Population-Based cervical Cancer screening: findings from Community-Based screening camps in Tamil Nadu, India. Asian Pac J Cancer Prev.

[41] Dhakal R, Noula M, Roupa Z, Yamasaki EN (2022). A scoping review on the Status of female breast Cancer in Asia with a special focus on Nepal. Breast Cancer (Dove Med Press).

[42] Parkin DM, Bray F, Ferlay J, Pisani P (2005). Global cancer statistics, 2002. CA Cancer J Clin.

[43] Bekos C, Schwameis R, Heinze G, Garner M, Grimm C, Joura E (2018). Influence of age on histologic outcome of cervical intraepithelial neoplasia during observational management: results from large cohort, systematic review, meta-analysis. Sci Rep.

[44] Solikhah S, Lianawati L, Matahari R, Rejeki DSS (2021). Determinants of breast Cancer screening practice among women in Indonesia: a Nationwide Study. Asian Pac J Cancer Prev.

[45] Islam RM, Billah B, Hossain MN, Oldroyd J (2017). Barriers to cervical Cancer and breast Cancer screening uptake in low-income and middle-income Countries: a systematic review. Asian Pac J Cancer Prev.

[46] Pfeiffer RM, Webb-Vargas Y, Wheeler W, Gail MH (2018). Proportion of U.S. Trends in breast Cancer incidence attributable to long-term changes in risk factor distributions. Cancer Epidemiol Biomarkers Prev.

[47] Karadag Arli S, Bakan AB, Aslan G (2019). Distribution of cervical and breast cancer risk factors in women and their screening behaviours. Eur J Cancer Care (Engl).

