# Supplementary figures and images for: Factors associated with positive cancer screening for the uterine cervix and breast in Jakarta Province, Indonesia: a cross-sectional study
Source: BMC Cancer. 2022 Dec 14;22:1309. doi: 10.1186/s12885-022-10381-1 (PMC9749285; doi:10.1186/s12885-022-10381-1)

## Slide 1
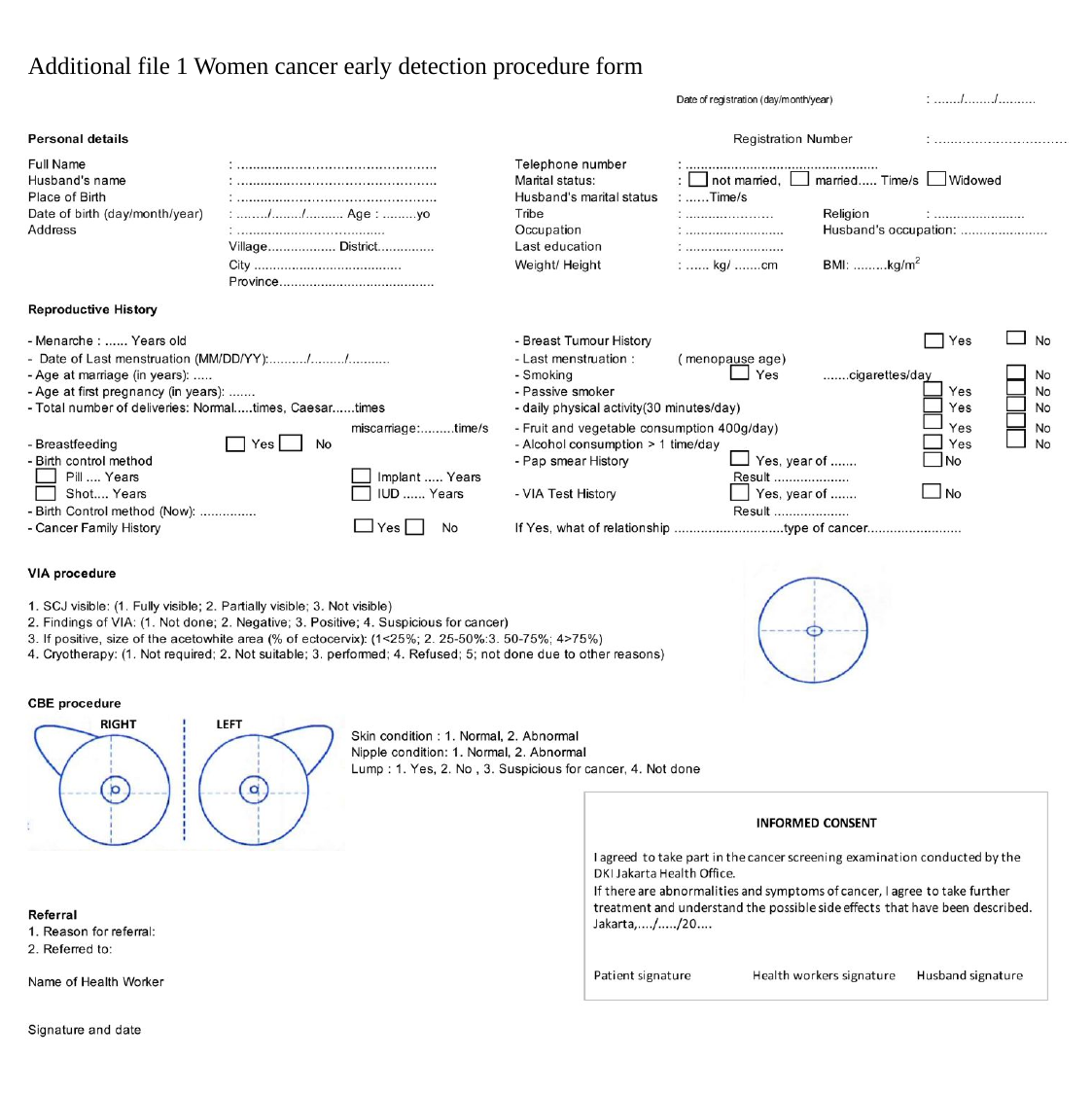

Additional file 1 Women cancer early detection procedure form

Supplement: Supplementary file 1 — Women cancer early detection procedure form. [file 12885_2022_10381_MOESM1_ESM.pptx]
